# Immunofluorescent detection of protein CoAlation in mammalian cells under oxidative stress

**DOI:** 10.1242/bio.061685

**Published:** 2024-09-30

**Authors:** Oksana Malanchuk, Antonina Khoruzhenko, Viktoriia Kosach, Anna Bdzhola, Dariy Bidiuk, Charlie Brett, Ivan Gout, Valeriy Filonenko

**Affiliations:** ^1^ Department of Cell Signalling, Institute of Molecular Biology and Genetics, National Academy of Sciences of Ukraine, Kyiv 03143, Ukraine; ^2^ Department of Structural and Molecular Biology, University College London, London WC1E 6BT, UK; ^3^ Department of Cell Screening, Enamine Ltd., Kyiv 02094, Ukraine; ^4^ Department of General Surgery, Danylo Halytsky Lviv National Medical University, Lviv 79000, Ukraine

**Keywords:** Coenzyme A, Protein CoAlation, Immunofluorescent analysis, Anti-CoA mabs, Cell signalling, Oxidative stress

## Abstract

Previously, we reported the generation and characterisation of highly specific anti-CoA monoclonal antibodies capable of recognizing CoA in various immunological assays. Utilizing these antibodies in conjunction with mass spectrometry, we identified a wide array of cellular proteins modified by CoA in bacteria and mammalian cells. Furthermore, our findings demonstrated that such modifications could be induced by oxidative or metabolic stress. This study advances the utility of anti-CoA monoclonal antibodies in analysing protein CoAlation, highlighting their effectiveness in immunofluorescent assay. Our data corroborates a significant increase in cellular protein CoAlation induced by oxidative agents. Additionally, we observed that hydrogen-peroxide induced protein CoAlation is predominantly associated with mitochondrial proteins.

## Teaser

**Summary:** The study demonstrates the effectiveness of anti-CoA antibodies in analysing protein CoAlation, particularly in immunofluorescent assay, and confirms a significant increase in protein CoAlation in response to oxidative stress.

## INTRODUCTION

Coenzyme A (CoA) is an essential cofactor involved in various biochemical reactions across all living organisms. It functions primarily as an acyl group carrier and a carbonyl-activating group. CoA and its thioester derivatives are crucial in numerous biosynthetic and degradative metabolic pathways, allosteric interactions, and gene expression regulation (Leonardi et al., 2005; Davaapil et al., 2014; Srinivasan and Sibon, 2014; Theodoulou et al., 2014).

CoA allocation in cellular compartments reflects its diverse roles in the cell with the highest concentration found in the mitochondria that 30-100 times higher than in the cytosol (Leonardi et al., 2005). Additionally, the size of the CoA pool (CoA and all its derivatives) varies widely among mammalian tissues, being the largest in the liver, heart, and kidney (Leonardi et al., 2005; Horie et al., 1986). The intracellular level of CoA and its derivatives are tightly regulated in response to extracellular stimuli, stresses, and metabolites, and varies in human pathologies, such as cancer, metabolic disorders, and neurodegeneration (Robishaw et al., 1982; Tubbs and Garland, 1964; Smith and Savage, 1980; Voltti et al., 1979; McAllister et al., 1988; Smith et al., 1978; Liu et al., 2019; Cavestro et al., 2023). Mutations in pantothenate kinase 2 (PANK2) and CoA synthase (CoASY), which are rate-limiting enzymes in the CoA biosynthetic pathway, are associated with a severe neurodegenerative disorder called NBIA (neurodegeneration with brain iron accumulation) (Zhou et al., 2001; Dusi et al., 2014).

The function of CoA in the cell is largely determined by the ability of the thiol group to form high-energy thioesters of CoA, and for a long time, studies have mainly focused on the participation of CoA and its derivatives in numerous metabolic processes in the cell. At the same time, the function of CoA as a low molecular weight (LMW) thiol in cells response to oxidative stress, as in the case of the most studied LMW thiol antioxidant glutathione, has not been investigated at all. The studies of possible antioxidant function of CoA were hampered by the absence of specific reagents and first of all anti-CoA antibodies.

Nonetheless, we have developed a highly specific anti-CoA monoclonal antibody, 1F10, which recognises CoA in various immunological assays (Malanchuk et al., 2015). The availability of such antibodies allowed us to demonstrate for the first time that CoA use the thiol group for covalent modification of oxidised cysteine residues under cell oxidative and metabolic stresses which we termed protein CoAlation (Tsuchiya et al., 2017).

Using an anti-CoA monoclonal antibody and mass spectrometry, we have identified a wide array of cellular proteins in bacteria and mammalian cells/tissues exposed to oxidative or metabolic stress (Tsuchiya et al., 2017, 2018).

Bioinformatic pathway analysis of identified proteins showed that they are preferentially implicated in major metabolic pathways, such as the Krebs cycle, β-oxidation, metabolism of glucose, etc., in which CoA and its derivatives are central cofactors and metabolites. In overall, about 65% of identified proteins to be CoAlated in response to the oxidative stress are implicated in cell metabolism (Tsuchiya et al., 2018). The susceptibility of metabolic enzymes to CoAlation in response to oxidative stress may reflect its potential regulatory role in the respond to the redox state (Filonenko and Gout, 2023).

We showed that protein CoAlation is a reversible posttranslational modification that is induced in bacteria and mammalian cells by oxidizing agents and metabolic stress (Tsuchiya et al., 2017, 2018). So far, the identity of enzymes responsible for the protein deCoAlation in mammalian cells is not known; however, recently we identified enzymes with such activity in bacterial cells (Tossounian et al., 2023).

Many key cellular enzymes were found to be CoAlated *in vivo*, with further *in vitro* studies demonstrating that this modification significantly affects enzymes activities differently than glutathionylation (Tsuchiya et al., 2017; Baković et al., 2019; Tsuchiya et al., 2020; Baković et al., 2021; Yu et al., 2021). Our study reveals that protein CoAlation is a widespread post-translational modification, which may play an important role in redox regulation under physiological and pathophysiological conditions.

To analyse the extent of protein CoAlation within different cellular compartments we introduced immunofluorescent analysis and subcellular fractionation for detection of CoA-modified proteins using anti-CoA mAbs.

## RESULTS

### Detection of anti-CoA immunospecific signal in non-cancerous cell lines

**Fig. 1.**
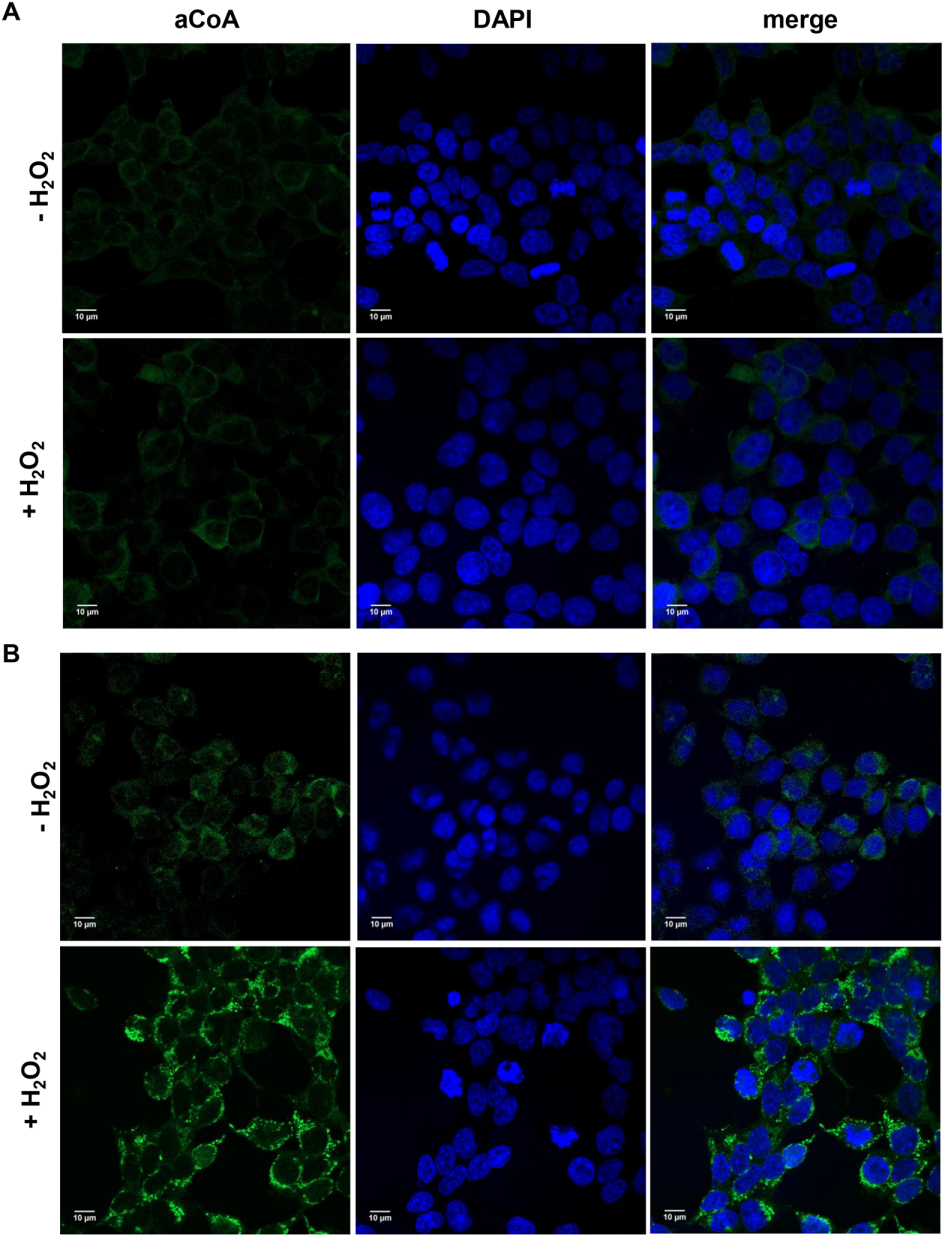
**IF detection of anti-CoA mAb immunoreactive signal in HEK293 (A) and HEK293/Pank1β (B) cells.** Cells were untreated or treated with 0.5 mM H_2_O_2_ before being fixed with formalin, permeabilised, and processed for immunofluorescence confocal microscopy using anti-CoA mAb (1F10) and FITC-anti-mouse secondary antibodies (green). DAPI (blue) was used to visualise the nuclei. Scale bars: 10 µm.

Immunofluorescent (IF) analysis of HEK293 and HEK293/Pank1β cells revealed an anti-CoA immunoreactive signal in both cell lines under normal cell growth conditions, with a significant enhancement in HEK293/Pank1β cells (Fig. 1). Stable overexpression of Pank1β, the major rate-limiting enzyme in the CoA biosynthetic pathway, results in significantly higher CoA levels in HEK293/Pank1β cells and, consequently, a more intense anti-CoA immunoreactive signal compared to HEK293 cells. Consistent with our previous western blotting (WB) analysis of H_2_O_2_-stressed HEK293/Pank1β cells (Tsuchiya et al., 2017), we observed a profound increase in protein CoAlation using IF (Fig. 1B). We also detected a moderate increase in the anti-CoA immunoreactive signal in H_2_O_2_-treated HEK293 cells (Fig. 1A). Overall, our data confirmed protein CoAlation in cells induced by oxidative agents that in addition is determined by the cellular level of CoASH, which varies across different tissues and cell lines (Tsuchiya et al., 2017).

### Detection of anti-CoA immunospecific signal in cancer cell lines

**Fig. 2.**
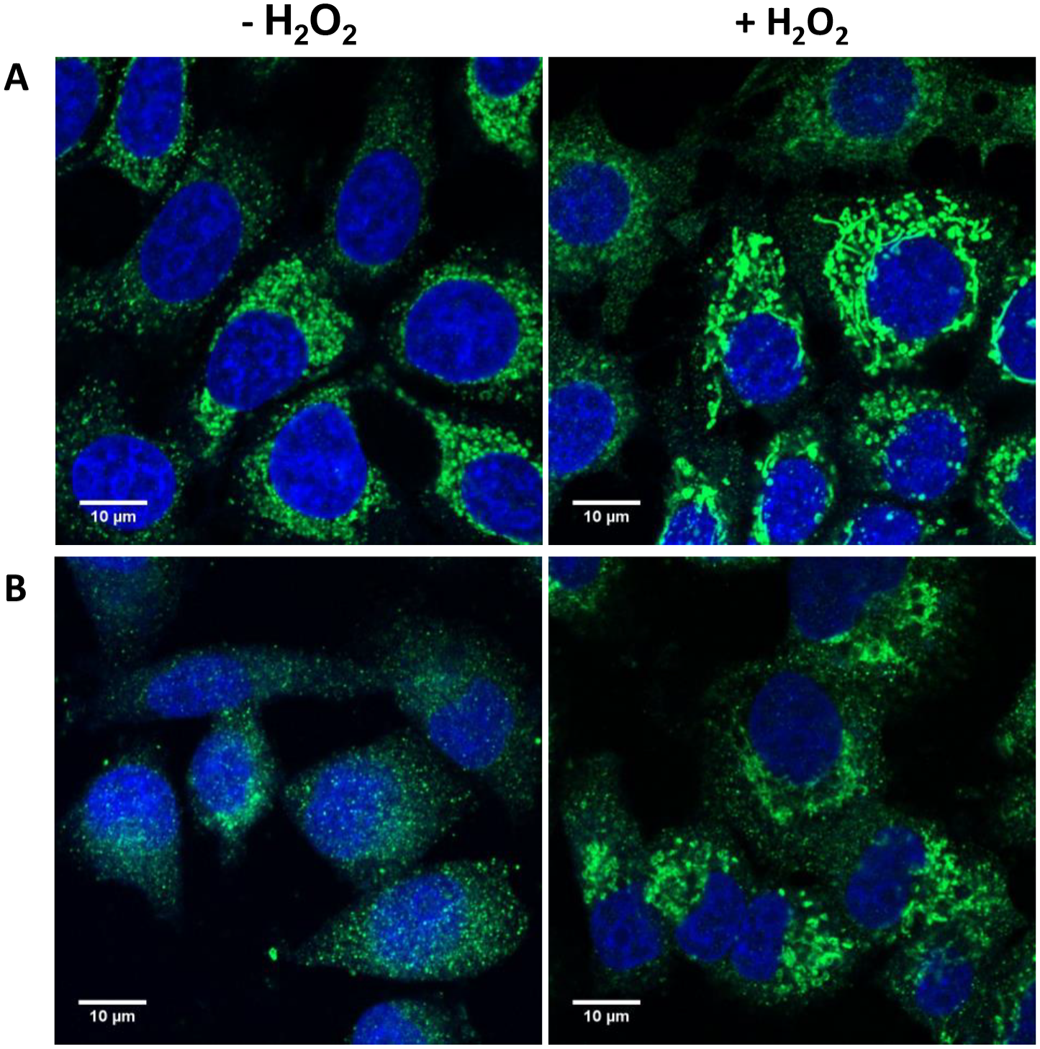
**IF detection of anti-CoA mAb immunoreactive signal in MCF7 (A) and MDA-MB-231 (B) cells.** Cells were untreated or treated with 0.5 mM H_2_O_2_ before being fixed with formalin, permeabilised, and processed for immunofluorescence confocal microscopy using anti-CoA mAb (1F10) and FITC-anti-mouse secondary antibodies (green). Nuclei were visualised by DAPI staining (blue). Scale bars: 10 µm.

Considering that the IF analysis of protein CoAlation, described above, was performed in model HEK293/Pank1β cells with artificially upregulated CoA level by Pank1β overexpression, we additionally conducted protein CoAlation analysis in unmodified cell lines. It is well-established that cancer cells undergo metabolic reprogramming and mostly rely on aerobic glycolysis to facilitate cell growth and metastasis (Warburg and Minami, 1923). Oncogenic transformation is accompanied by alterations in CoA and CoA-derivative concentrations, which may affect the levels of protein CoAlation. To investigate this, we selected cell lines from human tumour origins for analysis: the non-metastatic and metastatic breast adenocarcinoma cell line MCF7, and MDA-MB-231 correspondently. Based on further IF analysis, significant protein CoAlation was detected in both investigated cell lines even under normal conditions (Fig. 2, left panel). The induction of oxidative stress via H_2_O_2_ treatment significantly enhanced anti-CoA immunoreactive signal in both cancer cell lines (Fig. 2, right panel), as it was demonstrated previously for HEK293/Pank1β with elevated CoA expression level, but not for HEK293 cells with endogenous CoA level (Tsuchiya et al., 2017).

### Specificity analysis of immunofluorescent signals

**Fig. 3.**
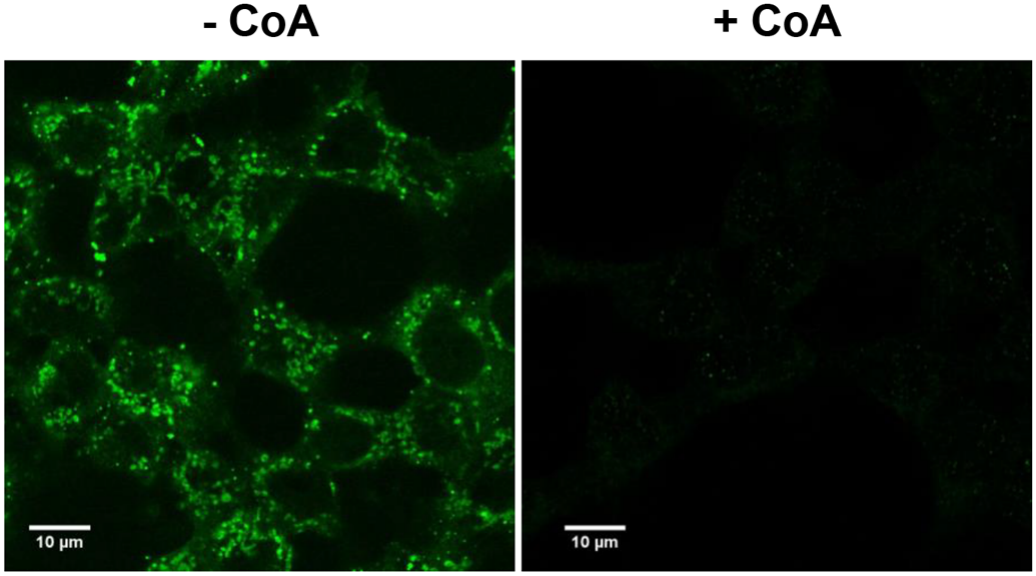
**IF detection of the anti-CoA mAb immunoreactive signal in H_2_O_2_-treated HEK293/Pank1β cells.** Formalin-fixed HEK293/Pank1β cells were incubated with primary anti-CoA mAbs (F10) in absence or presence of 10 mM CoA. Following this, immunofluorescence confocal microscopy analysis was performed using FITC-anti-mouse secondary antibodies (green). Scale bars: 10 µm.

The application of highly specific anti-CoA mAbs provides a unique approach for IF analysis to detect protein CoAlation under various physiological conditions, with the possibility to examine different cellular compartments. We conducted several experiments to confirm the specificity of the immunofluorescent signals generated by anti-CoA mAbs. To do this, we stained H_2_O_2_-stressed HEK293/Pank1β cells with anti-CoA mAbs depleted by free CoA and compared this to staining with undepleted antibodies (Fig. 3). According to our data, saturation of anti-CoA mAbs with free CoA decreases the intensity of immunofluorescent signals. This decrease in signal intensity confirms the specificity of the generated immunofluorescent signal to CoA-modified molecules (Fig. 3, left image).

**Fig. 4.**
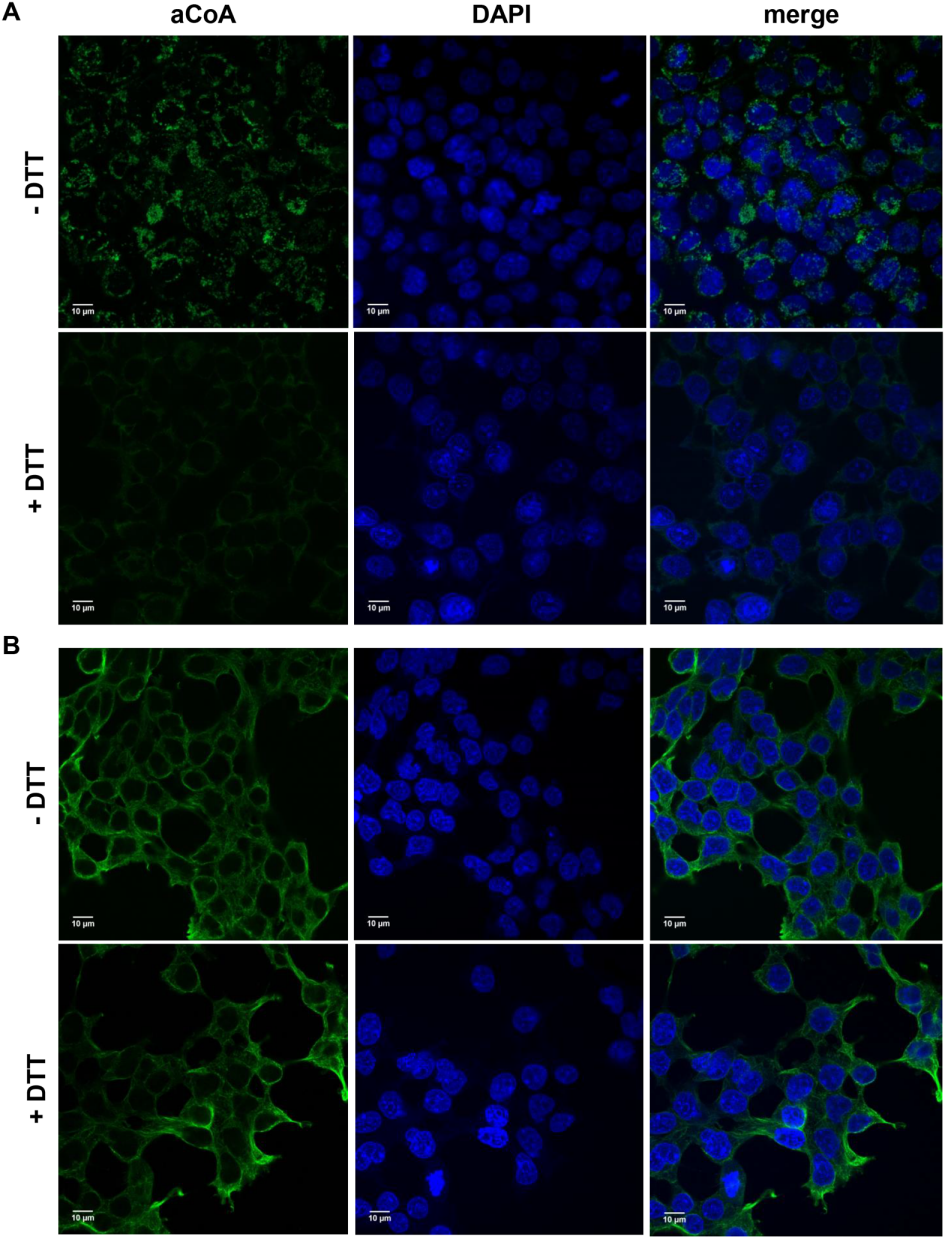
**Effect of DTT treatment of formalin-fixed cells on the intensity of anti-CoA and anti-β tubulin mAbs immunoreactive signals in H_2_O_2_-stressed HEK293/Pank1β cells.** Samples of HEK293/Pank1β formalin-fixed cells prior to incubation with anti-CoA mAbs (F10) (A) or anti-β tubulin mAbs (B) were incubated in PBS supplemented with 200 mM DTT with following immunofluorescence confocal microscopy analysis using FITC-anti-mouse secondary antibodies (green) and nuclear (blue) DAPI staining. Scale bars: 10 µm.

However, the above data does not consider the possibility of CoA-derivatives also being recognised in formalin-fixed cells along with CoA-protein mixed disulfides. Indeed, according to our data, anti-CoA mAbs (1F10) might not exclusively recognise CoA, but also succinyl-CoA, malonyl-CoA or glutaryl-CoA as well (Malanchuk et al., 2015). To clarify this issue, we introduced dithiothreitol (DTT) treatment of formalin-fixed cells before the incubation with anti-CoA mAbs, taking into consideration that protein CoAlation is reversible process that could be conversed by reducing agents (Tsuchiya et al., 2017). According to the presented data (Fig. 4A), DTT treatment significantly decreased anti-CoA mAbs signals in H_2_O_2_-treated cells. This result was expected, as under reducing conditions, CoA cannot form intra- or intermolecular disulfides with redox-sensitive cysteine residues, limiting the levels of protein CoAlation. At the same time the residual staining after DTT treatment may represent sites of free CoA and/or its derivatives within the cell in other types of complexes. Importantly, DTT treatment did not alter the efficacy of β-tubulin recognition by specific antibodies (Fig. 4B). Overall, this indicates that DTT treatment has no effect on the antigen-antibodies interaction and confirms the specificity of anti-CoA mAbs in the IF assay.

**Fig. 5.**
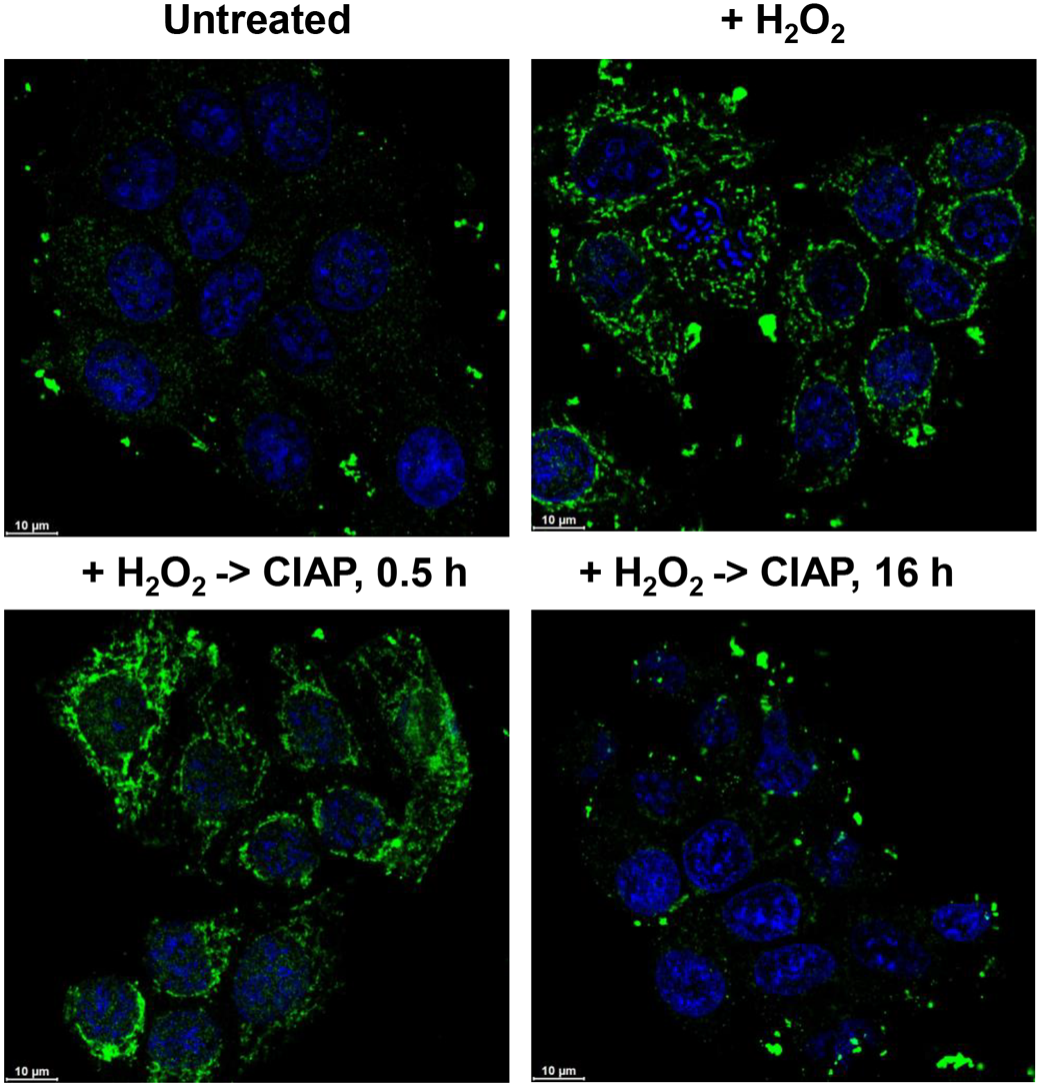
**Effects of CIAP treatment on H_2_O_2_-treated MCF7.** Formalin-fixed MCF7 cells prior to incubation with anti-CoA mAbs were treated with CIAP following H_2_O_2_ incubation within the indicated time period. Confocal microscopy analysis was then performed using Cy5-anti-mouse secondary antibodies (green) and nuclear (blue) DAPI staining. Scale bars: 10 µm.

Moreover, we have recently developed another highly specific anti-CoA monoclonal antibody (A11) using a different antigen preparation method (Malanchuk et al., 2022). Based on a specificity analysis that uses both types of anti-CoA mAbs (1F10 and A11), we concluded that CoA likely possesses a singular antigenic epitope, with the 3′-phosphate of the deoxyribose ring potentially serving as a key molecule in epitope formation for both antibody types. Therefore, based on this observation, we employed a dephosphorylation assay as another quality control of anti-CoA mAbs specificity. To do this, we initially induced oxidative stress by H_2_O_2_ in MCF7 cells and then treated the cells with 20 U of phosphomonoesterase CIAP to remove 3′-phosphates from 3'-phosphate 5'-diphosphate moiety of CoA and make the antigen determinant inaccessible for anti-CoA mAbs. Subsequent time-course analysis additionally confirmed our findings regarding the specificity of anti-CoA mAbs in the IF assay (Fig. 5).

### Mitochondrial localisation of anti-CoA mAbs immunoreactive signals

**Fig. 6.**
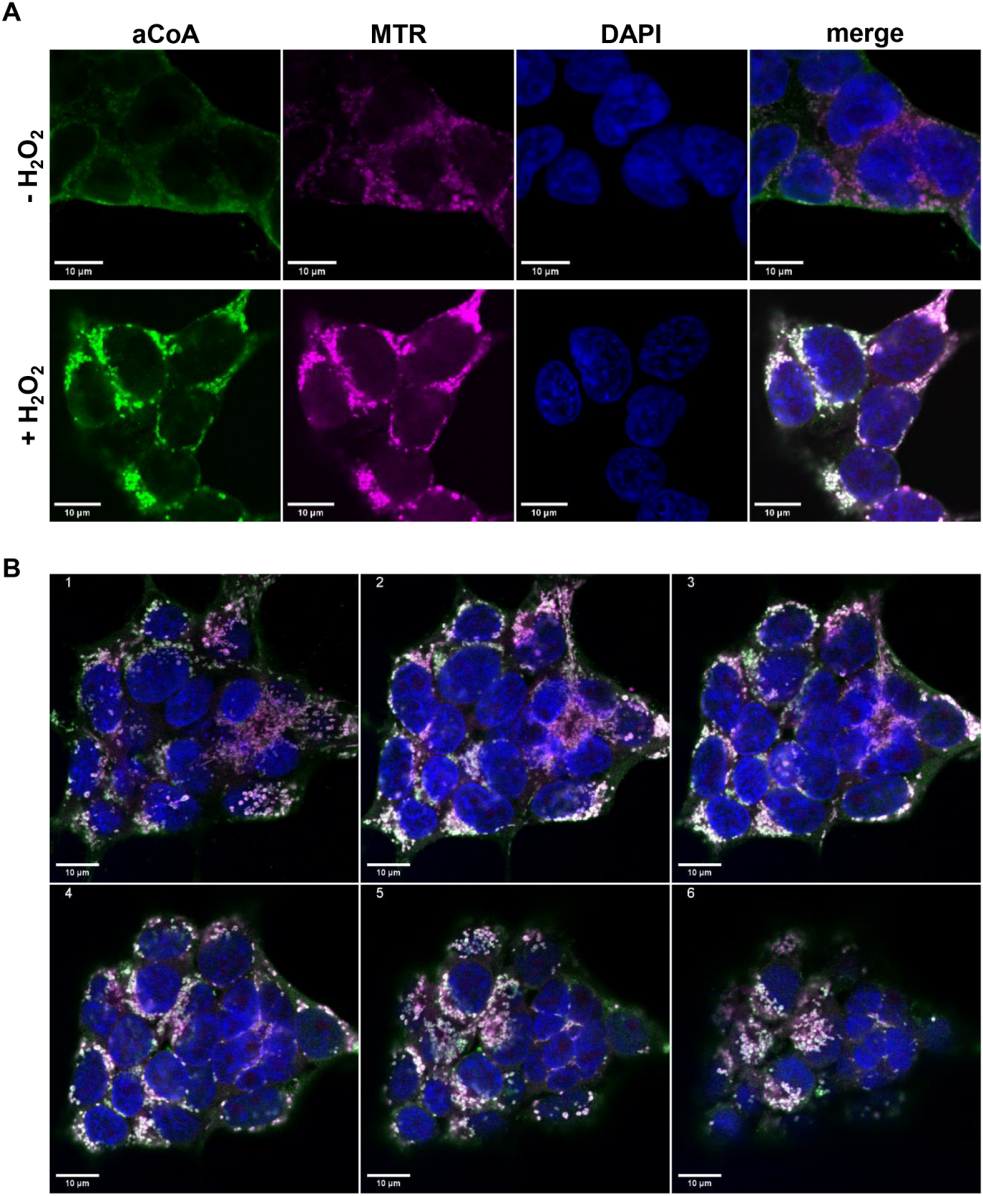
**Mitochondrial localisation of anti-CoA mAbs immunoreactive signals in H_2_O_2_ treated HEK293/Pank1β cells (A) with Z-stack imaging of merged anti-CoA and MitoTrackerTM Red CMXRos signals (B).** Cells were incubated with MitoTrackerTM Red CMXRos (red) before being fixed, permeabilised, and processed for confocal microscopy with the application of anti-CoA mAbs and FITC-anti-mouse secondary antibodies (green). Yellow in the overlay (merge) images appears where the green fluorescence of anti-CoA mAbs co-localises with the red fluorescence of MitoTracker. DAPI was used to visualise the nuclei (blue). Six representative optical sections with z-step of 1 µm and merged fluorescence signals are shown in B. The samples were imaged with a 63x oil objective lense (NA=1,4) using the Zeiss META 510 confocal microscope. Scale bars: 10 µm.

Analysis of the distribution patterns of CoAlated proteins detected by anti-CoA mAb IF signals across different cell lines led us to predict that this distribution is similar to that of cellular mitochondria (Figs 1-5). To verify this hypothesis, we labelled mitochondria with MitoTracker and CoAlated proteins with anti-CoA mAbs in HEK293/Pank1β cells, either under normal or H_2_O_2_-stressed conditions. Our data (Fig. 6) indicate that the IF signal intensity derived from anti-CoA mAbs demonstrate significant co-localisation with cellular mitochondria in H_2_O_2_-treated HEK293/Pank1β cells. This finding suggests an enrichment of CoA-modified proteins in the mitochondria during oxidative stress. These findings were further corroborated by analysis of subcellular fractions and performing Western blot analysis on H_2_O_2_-treated HEK293/Pank1β cells (Fig. S1). The specificity of detection was confirmed by applying both reducing and non-reducing WB conditions.

## DISCUSSION

Earlier, we generated highly specific anti-CoA monoclonal antibodies which recognise CoA and its derivatives, but not dephospho-CoA in various immunological assays (Malanchuk et al., 2015, 2022). The availability of an anti-CoA antibody enabled the development of mass spectrometry-based techniques, identifying about 2100 CoAlated proteins in eukaryotic and prokaryotic cells under conditions of oxidative and metabolic stress (Tossounian et al., 2022). Bioinformatic analysis of identified proteins has unveiled their involvement in various cellular processes, including intermediate metabolism, protein synthesis, muscle contraction, and stress response. Consequently, the above findings enabled us to investigate the subcellular localisation of CoAlated proteins in mammalian cells to estimate the extent of CoAlation within different cellular compartments. Previously we have demonstrated that the protein CoAlation level is determined by the cellular level of free CoA (CoASH) (Tsuchiya et al., 2017). In HEK293 cell lysates, the anti-CoA mAbs immunoreactive signal was hardly detected by WB. However, this signal was markedly increased in HEK293/Pank1β cells with stable overexpression of Pank1β, the main rate-limiting enzyme in CoA biosynthesis, which causes a significant elevation in cellular CoA levels (Tsuchiya et al., 2017). In addition, a strong increase in protein CoAlation was observed in HEK293/Pank1β cells, but not in HEK293 cells, under metabolic and oxidative stress conditions. These cells were exposed to metabolic stress via glucose deprivation, while oxidative stress was induced by the addition of cellular oxidants, including H_2_O_2_, diamide, menadione, TBH, etc.

The presented data corroborated our previous findings and clearly indicate that the mitochondrial fraction that represents of H_2_O_2_-stressed HEK293/Pank1β cells was enriched by CoAlated proteins. Furthermore, IF analysis of selected cancer cell lines, characterised by metabolic shifts and changes in CoA/CoA derivatives levels, which may impact protein CoAlation, indeed demonstrate strong anti-CoA immunoreactive signals even at normal cell growth conditions with the strong increase after oxidative agent treatment and showing the same distribution pattern of CoAlated proteins as in non-cancerous HEK293/Pank1β cells. The high level of protein CoAlation in cancer cells is probably related to the peculiarities of their metabolism and the redox balance. Functional analysis of protein CoAlation in cancerous cells will be the aim of our further studies.

Importantly, our IF data align with previous mass spectrometry (MS) results, suggesting that about 65% of CoAlated proteins are metabolic enzymes, with most being localised in the mitochondria that is a most CoA saturated cell compartment. In addition, we should emphasise that the MS data does not allow for quantitative analysis of CoAlated proteins. Rather, the WB and IF results clearly indicate that oxidative stress predominantly induces CoAlation in mitochondrial proteins. However, according to our recent data CoA-modified proteins could also be present in other cellular compartments. LC-MS/MS analysis of cells exposed to another oxidative agent – diamide revealed that the majority of CoAlated proteins are located in the cytoplasm, followed by the mitochondria (Tossounian et al., 2022). This can be explained by the different mechanisms of oxidative stress induction caused by diamide and hydrogen peroxide that requires further studies. Generally, the IF data confirmed the data of WB and MS analysis and demonstrated that the protein CoAlation is strongly influenced by oxidizing agents. In addition, we found significant basal levels of protein CoAlation that can be detected by IF, but not WB analysis in unstressed cells especially of cancer origin. These data suggest that CoA not only functions as a major cellular antioxidant to protect proteins from irreversible oxidative damage but might also be implicated in protein functions regulation by covalently modifying redox-sensitive cysteine residues and, therefore, could be a part of novel mechanism of response to the intracellular reactive oxygen species in context of their known signalling functions. It is also noteworthy that induced protein CoAlation predominantly occurs in the mitochondria. Experiments involving the treatment of cell samples with the reducing agent DTT confirm that anti-CoA mAbs detect protein CoAlation based on the formation of mixed disulfides and most likely not other CoA-dependent modifications.

The above data demonstrate that highly specific CoA mAbs are a powerful tool for further analysing the relevance of protein CoAlation in the functioning of mammalian cells.

## MATERIALS AND METHODS

### Mammalian cell culture

Different cell lines, including HEK293 (ATCC # CRL-1573), HEK293/Pank1β [cells stably overexpressing the rate-limiting enzyme in CoA biosynthesis, pantothenate kinase 1β (Tsuchiya et al., 2017)], MCF7 (ATCC #HTB-22), and MDA-MB-231 (ATCC #CRM-HTB-26), were cultured in Dulbecco's Modified Eagle Medium (DMEM) (Lonza), supplemented with 10% fetal bovine serum (FBS) (Hyclone), 50 U/ml penicillin, and 0.25 µg/ml streptomycin (Lonza). All cell lines were tested and shown to be free of mycoplasma infection.

### Treatment of mammalian cells

Mammalian cells were seeded onto 60 mm culture dishes (approximately 0.2-1 million) or cover glasses in 24-well plates (approximately 15,000 cells/well) and were allowed to grow for 24 h in complete DMEM with 10% FBS. The media was replaced with pyruvate-free DMEM supplemented with 5 mM glucose and 10% FBS, and the cells were incubated for another 24 h. Cells were then treated with H_2_O_2_ (0.5 mM) for 30 min at 37°C. When required, cells were additionally incubated with 20 U of calf intestinal alkaline phosphatase (CIAP; #18009019, Invitrogen, Thermo Fisher Scientific) for 0.5 h and 16 h. In certain experiments, samples were further incubated with PBS supplemented with 200 mM DTT at 60°C for 1 h, followed by numerous washings with PBS.

### Crude subcellular fractionation

Crude mitochondria-enriched (M), nuclear-enriched (N) and cytosolic (C) fractions were isolated from H_2_O_2_ treated HEK293/Pank1β cells (as described above) according to the Abcam protocol with some modifications. Briefly, HEK293/Pank1β cells plated on 10 cm dishes were washed once with PBS, resuspended in 0.5 ml of fractionation buffer (20 mM HEPES pH7.4, 10 mM KCl, 2 mM MgCl_2_, 1 mM EDTA, 1 mM EGTA, 100 mM NEM, protease and phosphatase inhibitor cocktail) and incubated 15 min on ice. Next, the cell suspension was passed through a 27-gauge needle 15 times, followed by centrifugation at 700 ***g*** for 5 min at 4°C. The pellet representing the nuclear-enriched fraction was resuspended using PBS with 0.1% SDS and sonicated to shear genomic DNA and homogenise the lysate. The collected supernatant was further separated by centrifugation at 10,000 ***g*** for 15 min at 4°C into the mitochondria-enriched pellet and cytosolic fraction. The mitochondrial pellet was processed as described for the nuclear pellet.

Total cell lysates (T) prepared from cell suspension were passed through a 27-gauge needle with following sonication. For WB analysis with anti-CoA 1F10 mAbs protein samples representing 1/100 or 1/20 part of total protein lysates (T, and C or M and N fractions correspondently) were applied.

### Western blotting

After appropriate treatments, cells were collected by pressure washing and centrifuged at 1800 ***g*** for 5 min at room temperature (RT). The media was removed, and cells were lysed in ice-cold Lysis Buffer (50 mM Tris/HCl, pH 7.5, 150 mM NaCl, 5 mM EDTA, 50 mM NaF, 5 mM Na_4_P_2_O_7_, 1% Triton X100) supplemented with protease inhibitor cocktail and 25 mM NEM. Lysates were centrifuged at 21,000 ***g*** for 5 min at 4°C, and the supernatants were collected. Samples of cell lysates containing 25 µg proteins were mixed with Laemmli buffer (final concentrations: 63 mM Tris/HCl pH 6.8, 10% glycerol, 2% SDS, 0.0025% Bromophenol Blue) with or without DTT (100 mM final) and boiled for 5 min at 95°C. Proteins were separated by SDS-PAGE (12%) and transferred to polyvinylidene fluoride (PVDF) membranes (Millipore). Membranes were blocked in TBST (50 mM Tris/HCl pH 7.5, 150 mM NaCl and 0.05% Tween 20) supplemented with 5% non-fat milk for 60 min at RT. They were then incubated for 2 h at RT or overnight at 4°C with 1F10 anti-CoA mAbs (1 µg/ml) and either rabbit anti-VDAC antibody (Cell Signalling Technology #4866, 1:4000 dilutions), or rabbit anti-PARP antibody (Cell Signalling Technology #9532, 1:6000 dilutions), or rabbit polyclonal anti-S6K1 C-terminal antibodies (1:4000 dilutions) have been previously described (Savinska et al., 2001), all diluted in TBST. After washing with TBST, membranes were incubated for 30 min at RT with goat anti-mouse IgG H&L-HRP (Jackson ImmunoResearch) diluted in TBST (1:10,000 dilutions). Then membranes were washed with TBST and then with TBS (without Tween 20) and protein bands were visualised using the ECL system (Millipore).

### IF microscopy

Mammalian cells were cultivated on cover glasses in complete DMEM with 10% FBS. The media was replaced with pyruvate-free DMEM supplemented with 5 mM glucose and 10% FBS, and cells were incubated for another 24 h. For mitochondria labelling in live cells, samples were incubated for 30 min at 37°C in medium supplemented with 0.05 mM MitoTrackerTM Red CMXRos (Invitrogen, M7512). Cells were then treated with oxidizing agents if needed. Cells were fixed with 10% neutral buffered formalin (Sigma, F5554) supplemented with 100 mM NEM (from 1 M stock in ethanol, Sigma, E3876) for 15 min at RT. Cell membrane permeabilization and antigen demasking were performed by adding 0.2% Triton X-100/PBS for 15 min at RT. To eliminate autofluorescence the samples were incubated for 15 min with 10 mM cupric sulphate and 50 mM ammonium acetate, pH 5.0. Non-specific binding of antibodies was blocked by incubating samples in PBS with FCS (10%) for 30 min at 30°C. Then samples were incubated with 1F10 anti-CoA mAbs (1:100 dilution) or mouse anti-β- tubulin mAbs (Sigma, T0198, 1:100 dilution) overnight at 4°C or 2 h at 30°C in a humidified chamber. The secondary FITC conjugated goat anti-mouse antibodies (Jackson ImmunoResearch Laboratories, Pennsylvania, USA) or the Cyanine5 conjugated goat anti-mouse IgG (H+L) cross-adsorbed secondary antibody (#A10524, Invitrogen, Thermo Fisher Scientific) were applied in dilution 1:400 (in 10% FBS/PBS) for 1 h at 30°C in a humidified chamber. The samples were washed with PBS and embedded into a Mowiol medium containing 0.1% DABCO and 0.1 mg/ml DAPI. Samples were imaged using the Zeiss META 510 confocal microscope with either a 63× (NA=1,4) or 40× objective lens (NA=1,3). The Leica TCS SPE confocal system was also used. All specimens within the same experiment were acquired at the same intensity/exposure/laser power parameters to ensure that the signal intensities could be adequately compared and represented. For Z-stack acquisition nine optical slices were taken with an optical section separation (Z-interval) of 1 µm using 63× oil objective lens (NA=1,4) and Zeiss META 510 confocal microscope. The samples were excited with 405-, 488-, and 543-nm laser lines, and emission was captured with a Zeiss META detector.

## Supplementary Material

10.1242/biolopen.061685_sup1 Supplementary information
