# Supplementary material for: Immunofluorescent detection of protein CoAlation in mammalian cells under oxidative stress
Source: Biol Open. 2024 Sep 30;13(10):bio061685. doi: 10.1242/bio.061685 (PMC11463958; doi:10.1242/bio.061685)
Supplement: Supplementary information [file biolopen-13-061685-s1.pdf]

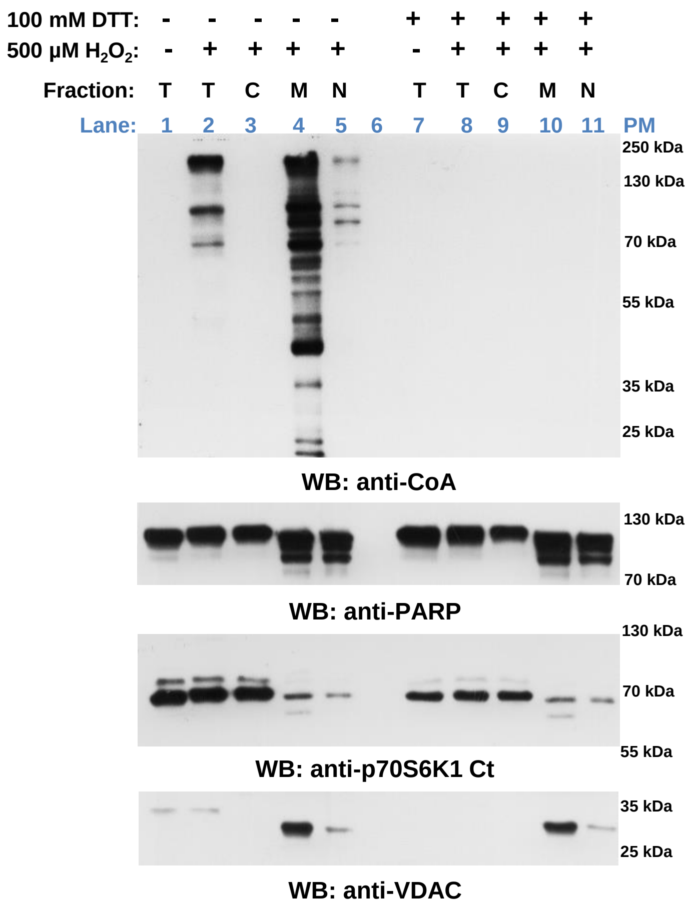

Fig. S1. Subcellular fractionation of CoAlated proteins in H_2O_2-treated HEK293/Pank1β cells. HEK293/Pank1β cells after treatment with H_2O_2 were disrupted into crude cell lysate (T) and separated into cytosol (C) and fractions enriched by nuclei (N) and mitochondria (M) as described under "Materials and Methods". Equal parts of T, C and M, N fractions (1/100 and 1/20 correspondently) were resolved by SDS/PAGE in the presence or absence of DTT in loading buffer and immunoblotted with anti-CoA mAbs (1F10). Ribosomal protein S6 kinase 1 (S6K1), VDAC and PARP were used as markers of cytosolic, mitochondrial, and nuclear fractions correspondently.

Biology Open • Supplementary information
